# Evaluation of matrix-assisted laser desorption ionisation time-of-flight mass spectrometry (MALDI-TOF MS) for the Identification of Group B *Streptococcus*

**DOI:** 10.1186/s13104-019-4119-1

**Published:** 2019-02-14

**Authors:** Ka-Ning To, Emma Cornwell, Roger Daniel, Sweenie Goonesekera, Elita Jauneikaite, Victoria Chalker, Kirsty Le Doare

**Affiliations:** 1 Section of Paediatrics, Department of Medicine, Imperial College London, London, UK; 2 Centre for Infection and Immunity, St George’s University of London, London, UK; 3 Public Health England, London, UK; 4 Infection and Immunity, Department of Microbiology, Charing Cross Hospital, London, UK; 5 Department of Infectious Disease Epidemiology, Imperial College London, London, UK

**Keywords:** Group B *Streptococcus*, *Streptococcus agalactiae*, Rapid diagnostics, MALDI-TOF MS

## Abstract

**Objective:**

Group B *Streptococcus* (GBS) is a leading cause of neonatal meningitis and sepsis worldwide. Intrapartum antibiotics given to women carrying GBS are an effective means of reducing disease in the first week of life. Rapid and reliable tests are needed to accurately identify GBS from these women for timely intrapartum antibiotic administration to prevent neonatal disease. Many laboratories now use matrix-assisted laser desorption ionisation time-of-flight mass spectrometry (MALDI-TOF MS) by direct plating or cell lysis for the identification of GBS isolates. The cell lysis step increases time to results for clinical samples and is more complex to perform. Therefore, we seek to evaluate the sensitivity and specificity of the quicker and more rapid direct plating method in identifying GBS.

**Results:**

We directly compared swab isolates analysed by both direct plating and cell lysis method and demonstrated that direct plating has a sensitivity and specificity of 0.97 and 1, respectively, compared to an additional cell lysis step. We demonstrated that MALDI-TOF MS can be successfully used for batch processing by the direct plating method which saves time. These results are reassuring for laboratories worldwide who seek to identify GBS from swabs samples as quickly as possible.

## Introduction

Group B *Streptococcus* (GBS or *Streptococcus agalactiae*) is a Gram-positive bacterium found colonising in the genitourinary and gastrointestinal tracts of approximately 20% of pregnant women [1–3]. Maternal colonisation is the primary source of transmission in neonatal GBS infections as the bacterium can spread in utero*,* or from neonatal inhalation or ingestion of contaminated vaginal fluids. Infections can cause still- and preterm-births, foetal sepsis, meningitis, pneumonia and neurodevelopmental complications in survivors of GBS meningitis [3, 4]. Intrapartum antibiotic prophylaxis (IAP) can be given to pregnant women in labour to prevent neonatal early-onset disease if GBS has been identified by swab-based screening. However, culture-based methods of GBS detection can require a long turnaround time of 3–5 days [5] from swab being taken to results being given, and a number of women will not know their diagnosis before going into labour and will therefore not receive timely IAP.

Public Health England guidelines recommend inoculating swabs in a selective enrichment broth prior to sub-culturing specimen on blood or chromogenic agar plates to detect GBS carriage [6]. Presumptive GBS colonies from agar plates should then be confirmed through antigen-specific tests, biochemical tests and/or matrix-assisted laser desorption ionisation time-of-flight mass spectrometry (MALDI-TOF MS) [7]. Antigenic or biochemical tests are less favourable than MALDI-TOF MS as they offer lower sensitivity [5] and have been shown to cross-react to give false identification [8]. MALDI-TOF MS is quick, sensitive and economical. Currently, there are two sample preparation methods for MALDI-TOF MS analysis: direct plating or a full protein extraction by cell lysis [9, 10]. The direct plating method is considered to be less complex and more rapid than the cell lysis method. MALDI-TOF MS is 100% accurate for β-haemolytic *Streptococcus* [11], however Bizzini et al., showed that MALDI-TOF MS identification accuracy can be influenced by preparatory methods used [12]. The use of and reliance on the accuracy of identification by MALDI-TOF MS is increasing in clinical laboratories globally [13], with Food and Drug Administration approval for MALDI-TOF MS systems and time savings well documented [14]. It is important to ascertain the optimal methodology for GBS species identification for laboratories considering the increased use of MALDI-TOF MS. In this study we describe the largest investigation of GBS isolates to date to inform microbiology laboratories guidance in the identification of GBS comparing direct plating and cell lysis MALDI-TOF MS preparatory methods.

## Main text

### Methods

#### Bacterial isolates

947 *Streptococcus*, *Lactococcus*, *Aerococcus* and *Weissella* spp. isolates were cultured from clinically diverse rectal, vaginal and nasopharyngeal swabs in skim milk, tryptone, glucose, and glycerin (STGG) from pregnant women and infants [15]. To culture the bacteria, 200 µl of STGG was inoculated in 2 ml LIM RambaQUICK StrepB (CHROMagar, France) and incubated at 37 °C in 5% CO_2_ for 6–24 h. 10 µl of overnight growth was plated on CHROMagar StrepB (CHROMagar, France) at 37 °C in 5% CO_2_ for 18–24 h. Presumptive GBS colonies with a mauve morphology were selected to undergo a confirmatory test by MALDI-TOF MS. Bacteria plated on Columbia blood agar (Oxoid, England) were incubated at 37 °C in 5% CO_2_ for 18–24 h.

#### MALDI-TOF MS preparation

Confirmation of bacterial species identification using MALDI-TOF MS were performed as previously described [11]. Briefly, for the direct colony method, a single colony was picked and spotted onto a steel target plate (Bruker Daltonics, Germany) and allowed to dry before overlaying with 1 µl of MALDI matrix (Bruker Daltonics, Germany), a solution of α-cyano-4-hydroxycinnamic acid (HCCA) dissolved in 50% acetonitrile and 2.5% trifluoroacetic acid. For the cell lysis method, 1 µl loopful of bacteria was taken and suspended in 300 µl of molecular grade water and vortexed. 900 µl of 100% ethanol was added, vortexed then centrifuged at 16,000*g* for 2 min. The supernatant was discarded and the pellet left to dry at room temperature. Next, the pellet was resuspended in 30 µl of 70% formic acid and 30 µl of 100% acetonitrile then centrifuged at 16,000*g* for 2 min. 1 µl of supernatant was spotted onto the target plate and 1 µl of MALDI matrix directly overlaid. MALDI-TOF MS was performed using MALDI Biotyper (Bruker Daltonics, Germany) according to manufacturer’s recommendations. Spectra were analysed using Bruker Biotyper software (version 4.1.70). Using the manufacture’s criteria, log scores ≥ 2.00 indicate identification to species level, scores 1.70–1.99 indicate genus identification, and scores < 1.70 were interpreted as unreliable and were repeated. Isolates were tested in duplicate and deemed reliable and correct if identification of the repeated sample was the same species.

#### API testing

API biochemical test strip kit (bioMérieux, USA) was used to identify the bacteria species of the nine discordant results. The test was performed following manufacturer’s instructions. Results from the tests were analysed through APIWEB software (bioMérieux, USA).

#### Statistical analysis

Comparisons of genus- or species-level identification using direct plating versus protein extraction method were made using McNemar’s paired test on GraphPad Prism (version 7.03). *P* value < 0.05 were considered statistically significant.

### Results

#### MALDI-TOF MS sample preparation methods

We compared the direct plating method against a cell lysis method for MALDI-TOF MS analysis on 96 colonies that exhibit similar morphologies to GBS on CHROMagar from a sub-set of 33 clinically diverse swabs collected from mother-infant pairs. All isolates were identified to the genus level (log score 1.70–1.99) using either of the two methods. Cell lysis was able to identify 91/96 isolates to the species-level (log score ≥ 2.00) and direct plating identified 88/96 (Table 1). Isolates were correctly identified by both methods as *Streptococcus agalactiae* (*n* = 36), *Streptococcus salivarius* (*n* = 1), *Weissella confusa* (*n* = 2), *Lactococcus garvieae* (*n* = 45), *Lactococcus lactis* (*n* = 8) and *Aerococcus viridans* (*n* = 4). The sensitivity and specificity for direct plating compared to cell lysis were 0.97 and 1, respectively. Positive and negative predictive values for this method were 1 and 0.99, respectively.

**Table 1:** MALDI-TOF MS identification of *Streptococci*, *Lactococci*, *Weisella* and *Aerococci* to genus- and species-level by direct plating and cell lysis preparatory method

Organism	Number of isolates	Direct plating		Cell lysis	
		Genus-level identification only	Species-level identification	Genus-level identification only	Species-level identification
*Streptococcus agalactiae*	36	1	35	0	36
*Streptococcus salivarius*	1	1	0	0	1
*Weissella confusa*	2	0	2	0	2
*Lactococcus garvieae*	45	6	39	5	40
*Lactococcus lactis*	8	0	8	0	8
*Aerococcus viridans*	4	0	4	0	4
Total	96	8	88	5	91

#### Identification of GBS using CHROMagar and direct plating MALDI-TOF MS method

**Fig. 1:**
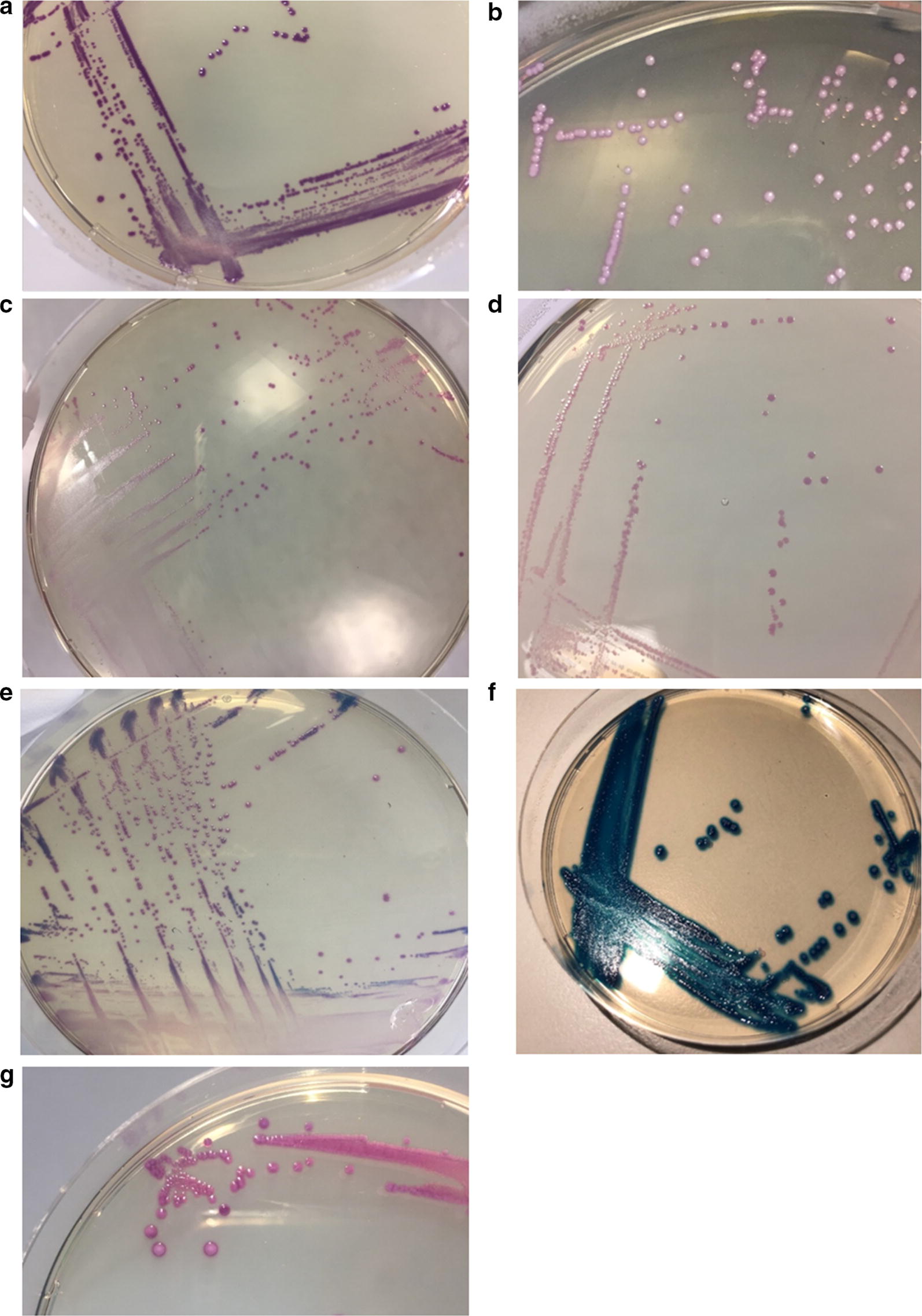
Microbiological colony appearance on CHROMagar after 18–24 h incubation. **a*** Lactococcus garvieae*; **b*** Aerococcus viridans*; **c*** Weissella confusa*; **d*** Lactococcus lactis*; **e*** Streptococcus salivarius; ***f*** Entercoccus faecalis* and **g** Group B *Streptococcus*

We analysed 851 rectovaginal, rectal and nasopharyngeal swabs from 155 mother-infant pairs using the direct plating method for MALDI-TOF MS analysis. These swabs had initially been identified as GBS positive by Columbia blood agar and confirmed using MALDI-TOF MS by direct plating. 842 (98.9%) isolates had the typical morphology of GBS and 9 (1.4%) isolates had morphologies not suggestive of the typical GBS appearance. The nine discordant samples had similar colony morphologies, with different colouration in some cases, to GBS on CHROMagar (Fig. 1) and were identified as GBS by the direct plating method on MALDI-TOF MS. These discrepant strains were subsequently re-analysed by API biochemical test strip kits and MALDI-TOF MS by cell lysis method as previously described [10], and were confirmed as *Weissella confusa* (*n* = 4), *Streptococcus salivarius* (*n* = 2), *Aerococcus viridans* (*n* = 2) and *Enterococcus faecalis* (*n* = 1).

### Discussion

To our knowledge, this is the largest study evaluating MALDI-TOF MS preparation methods for GBS identification from clinical specimens in combination with CHROMagar. Previous studies have demonstrated MALDI-TOF MS to be a valuable tool for identification of GBS with a high level of accuracy [10, 16, 17].

Several species such as *Enterococcus* spp.*, Streptococcus bovis, Streptococcus porcinus, Streptococcus pseudoporcinus, Streptococcus salivarius, Streptococcus thoraltensis, Streptococcus anginosus, Streptococcus pyogenes* and *Staphylococcus *spp. have been documented to develop colonies that resemble GBS on chromogenic agar compared to blood agar [5]. In our study, we found that MALDI-TOF MS incorrectly identified 9 isolates that had morphology similar to GBS on CHROMagar. However, when we compared direct plating with cell lysis, we found no difference in either approach to accurately identify GBS.

MALDI-TOF MS is a powerful tool for microbial identification, however, for some microorganisms this instrument is unable to discriminate between genus and species due to lack of reference spectra [18, 19], low identification scores [20] or low resolution of phylogenetically similar species [1]. Poor sample preparation can also influence the quality of results and care must be made to ensure sample and matrix are air-dried to prevent liquid smears and cross-contamination between spots on the target plate [12]. As MALDI-TOF MS spectra rely on the ionisation of proteins on the bacterial surface, some authors suggest that an additional cell lysis step is required for the species-level identification of Gram-positive bacteria to lyse the peptidoglycan cell wall and enable unmasking of these surface proteins to increase the protein profile [12]. This is supported by this study in which increased species level detection was achieved using cell-lysis as opposed to direct plating. Some *Streptococcus* species such as *S. viridans* [21], *S. pneumoniae* [22], and *S. mitis* [23] have been mis-identified through direct plating MALDI-TOF MS methods due to similarities in protein mass spectra. However, in our study GBS was correctly identified by both methods.

Evaluation of both preparatory methods have been studied in smaller studies for GBS [10, 16, 24, 25] demonstrating that direct plating gives high resolution in assigning species identification. In our study, we confirm that direct plating gives an accurate identification of GBS and species that resembled GBS on CHROMagar, without the requirement of a cell lysis extraction.

To conclude, the use of MALDI-TOF MS using direct plating preparatory methods is a reliable way to accurately identify GBS in a clinical diagnostic laboratory. A marginal increase in species level detection can be achieved using cell lysis but requires considerable extra effort over and above direct plating and has no overall effect on the specificity of GBS identification. Accurate bacterial species identification is key to the timely administration of IAP and can directly influence clinical care in cases with GBS that can be facilitated by MALDI-TOF MS [5]. The direct plating method is less time-consuming for large sample numbers as the application takes minutes whilst the cell lysis procedure can take half a day when batch processing. This makes direct plating more favourable in hospital diagnostic settings where rapid results are critical to determine whether a patient requires time-critical treatment, such as correctly identifying a woman requiring intrapartum antibiotic prophylaxis to prevent transmission of GBS, or to determine the best course of treatment in the case of infant GBS meningitis or sepsis.

## Limitations

Although we cannot explain the reason for the nine discordant results found, API biochemical tests confirmed the isolates to be non-GBS. Since we have shown that both MALDI-TOF MS preparatory methods consistently gave reliable identification of GBS, the reason for the discordance requires further investigation. It could reflect either lack of purity of colonies on agar plate, storage of the inappropriate strain or a potential contamination during sample preparation on the target MALDI-TOF MS plate.
